# Combining sentiment analysis classifiers to explore multilingual news articles covering London 2012 and Rio 2016 Olympics

**DOI:** 10.1007/s42803-022-00052-9

**Published:** 2022-11-16

**Authors:** Caio Mello, Gullal S. Cheema, Gaurish Thakkar

**Affiliations:** 1 School of Advanced Study, University of London, London, United Kingdom; 2 TIB - Leibniz Information Center for Science and Technology, Hannover, Germany; 3 Faculty of Humanities and Social Sciences, University of Zagreb, Zagreb, Croatia

**Keywords:** Olympic legacy, Sentiment analysis, NLP, News articles, Explainable AI

## Abstract

This study aims to present an approach for the challenges of working with Sentiment Analysis (SA) applied to news articles in a multilingual corpus. It looks at the use and combination of multiple algorithms to explore news articles published in English and Portuguese. It presents a methodology that starts by evaluating and combining four SA algorithms (SenticNet, SentiStrength, Vader and BERT, being BERT trained in two datasets) to improve the quality of outputs. A thorough review of the algorithms’ limitations is conducted using SHAP, an explainable AI tool, resulting in a list of issues that researchers must consider before using SA to interpret texts. We propose a combination of the three best classifiers (Vader, Amazon BERT and Sent140 BERT) to identify contradictory results, improving the quality of the positive, neutral and negative labels assigned to the texts. Challenges with translation are addressed, indicating possible solutions for non-English corpora. As a case study, the method is applied to the study of the media coverage of London 2012 and Rio 2016 Olympic legacies. The combination of different classifiers has proved to be efficient, revealing the unbalance between the media coverage of London 2012, much more positive, and Rio 2016, more negative.

## Introduction

Sentiment Analysis (SA) is a Natural Language Processing (NLP) technique to identify emotions discursively expressed in a text (Liu, 2012). Also called Opinion Mining (Feldman, 2013), it has been strongly associated with product reviews, to track feedback shared by consumers through comments on a website (Dave et al., 2003; Balahur, 2013), or social media content like Twitter (Pak & Paroubek, 2010).

This work aims to apply Sentiment Analysis algorithms to study news articles. It looks at the news media coverage of the legacies of two events: London 2012 and Rio 2016 Olympic Games. Different approaches aiming to deal with the specificities of news articles were raised by authors such as Balahur (2013), proposing a distinction between author, reader and text opinions; Nguyen et al., (2017) developing an aspect-level approach to handle with the long length of texts; and Shirsat et al., (2017) using document-level SA to compare sentiment among different news sections such as sports and politics.

This journalistic text genre presents specificities in form and content such as the length of the text, the minimised use of adjectives and the diversity of topics covered in one article, that require some effort to adapt traditional approaches projected for the use in subjective statements (Shirsat et al., 2017; Balahur, 2013; Taj et al., 2019).

Many challenges of working with these algorithms, like negation, when *not* precedes a noun, were addressed by Hussein (2018). Language is, however, one of the most important limitations. Although different techniques to work with non-English text have been developed (Dashtipour et al., 2016), many approaches have relied on machine translation to transform the texts into English before using the sentiment analysis algorithms (Balahur 2012). By translating the source language corpus into the target language, Machine Translation (Wan, 2009; Wei & Pal, 2010) has been utilised to solve the issue of cross-lingual sentiment analysis. Banea et al., (2008) translated a corpus into the target language and classified the data using the classifier in the source language. Wan (2008), proposed combining resource translation of source and target data with ensemble learning in order to train a bilingual classifier. Aside from simple training data translations, attempts have been made to bridge the language barrier using parallel unlabeled data (Meng et al., 2012; Lu et al., 2011), using translation services in conjunction with monolingual and bilingual constraints, and generating sentiment lexicons (Mohammad, Salameh & Kiritchenko, 2016) for languages with limited resources.

This work applies both algorithms trained in Portuguese and translation into English. Machine Translation approaches can be a problem for languages without reasonable translation modules, where mistakes can lead to wrong sentiment outputs. For Portuguese, studies suggested that although errors occur, automated translation is a decent approach (Araújo et al., 2020; Pereira, 2021; De Freitas & Vieira, 2015).

Besides analysing these events by applying sentiment classifiers to the texts, this paper also reflects on the potentialities and limitations of using this technique. As stated by Mannarswamy & Chidambaram (2021), NLP techniques can be referred to as black boxes. From the perspective of the Digital Humanities, not knowing what is behind the outputs provided by an algorithm can lead to questions about the validity of the method, impacting the analysis. To understand the algorithms used in this work, we make use of explainable AI (Došilović et al., 2018). This concept refers to the exercise of looking at the way these algorithms work and explaining their results. The explainability and interpretability methods (Linardatos et al., 2021; Molnar, 2022) rely on certain assumptions, and the outputs of these methods should be understood from an explorative point of view. The methods discussed in this paper do not point to how the sentiment classifiers work and behave but only suggest what the underlying models might be looking at (or using for) the prediction they make. This helps with trust in the results from a Digital Humanities perspective, as the data will be used to infer conclusions about the phenomenon represented in the case study.

## Case Study: the olympic legacy of London 2012 and Rio 2016

Every four years, a different city becomes the centre of attention for hosting the biggest sporting event in the world: the summer Olympic Games. Along with the spotlights that allow countries to communicate their agenda globally, comes the eyes of the media to watch closely the benefits and negative impacts of these investments. These consequences of the Olympic Games are officially called ‘legacies’. Although highly associated with a positive meaning, the word legacy describes not only the gains of the cities by hosting the event but all the aspects that emerge from this process, whether ‘planned or unplanned, positive or negative, intangible or tangible’ (Gratton & Preuss, 2008, p. 1924).

The number of bids to host the Olympic Games has reduced over time. While for the 2004 Olympics, there were 11 cities competing to host the event, in the recent 2022 and 2024 bids, there were only two^3^ and three^4^ respectively, because most of the initial candidates withdrew from the bidding process. In this scenario where hosting the Olympics has become less attractive due to cost rising, reduction in profit from TV licensing among other aspects, Olympic legacy becomes even more crucial for the continuation of the games as it is part of a ‘legitimating narrative’ (Poynter & MacRury, 2009, p. 315). For Gratton & Preuss (2008), ensuring a positive legacy is fundamental as it prevents people from blaming the International Olympic Committee (IOC) after the event, it justifies the use of scarce public resources, and it also encourages other cities to bid for future events.

Understanding the legacy of the Olympics is not just important for the IOC but also for the cities to evaluate their past decisions and plan the future. Legacy as a concept is often used to express the desire of a utopian city to be achieved by using the Games as a development catalyst (Girginov, 2018, p.196). The utopian thought has been crucial in urban planning and has highly contributed to the transformation of urban landscapes worldwide (Pinder, 2002). At the same time as news like *Six years later, the wonderful legacy of London 2012* (Pussieldi, 2018) and ‘London and its Olympic legacy: East-side of London still growing 5 years after the games’ (Dilascio, 2017), published by Globo, are highlight the positive impact of the Games, others focus on negative effects.

*Rio’s Olympic legacy a ‘huge disappointment’* (Davies, 2017) and *Key London Olympic legacy ‘a failure’, says Tessa Jowell* (BBC, 2015) are examples of news titles that emphasise negative legacies of the Olympics. The contrast between positive and negative news articles emerges in the middle of a dispute for the meaning of the concept of ‘legacy’. While activists raised their concerns on how these events are planned and executed, governments reinforced their quality management to deliver a successful set of legacies. Other entities such as the IOC also participated in the dispute, claiming its importance as facilitators or channels for the delivery of the legacy. For this study, we focused on the role of news outlets for the construction of the Olympic legacy narrative.

## A media event

Daily news not only informs the audience of events that happened, but they also construct the events. Newspapers, television, radio and news websites decide on where the attention of the public should be focused by ranking the articles according to their ‘relevance’. The role of organising the events in terms of ‘news values’ is called agenda-setting (McCombs, 2004). Through this process of gathering and formatting information, journalism organises the world and becomes a ‘place of reference’ Vizeu (2009, p.77). It is a ‘place’ where people ‘go’ in search of stability, as it helps to interpret the world.

In the appendix of his book ‘On Television’, Bourdieu (1998) highlights the discursive aspect of the Olympic Games, as he argues that the event is produced twice: first in the stadium, and second in images and commentary (Bourdieu, 1998, p.81). This understanding that the media not only mediates but actively constructs the narrative is fundamental. As a media event, the news articles covering the Olympic legacy are read here as a place of reference, as they provide readers with an interpretation of facts to be communicated and reinterpreted by an active audience.

Based on this understanding, the method of Sentiment Analysis will be used to explore the positive and negative utterances quantitatively in news articles covering two events: London 2012 and Rio 2016 Olympic Games. The main purpose is to find clues to understand how the media addresses the legacy of these events allowing comparisons over time, over news outlets and over two events that happened in contrasting social-political-geographical and economic contexts.

## Methodology

The methodology of this research was divided into three steps: data collection, data preparation and development of an analytical method using sentiment detection techniques. Each of them will be described in detail in the following sections.

## Data collection

The data is composed of 1271 news articles scraped from seven media outlets published in Portuguese and English covering the Olympic legacy of London 2012 and Rio 2016. Of the seven media outlets, four are British (BBC, The Guardian, Daily Mail, and The Telegraph), and three are Brazilian (Globo, Folha de S. Paulo and Estadao)^5^. BBC and Globo are the British and Brazilian broadcast companies that held the TV licence of the Olympics in 2012 and 2016. They are also the most accessed online news websites respectively in the United Kingdom and Brazil. Daily Mail, The Telegraph, The Guardian, Folha de S. Paulo and Estadao are the most accessed websites of newspapers in the United Kingdom, according to the Ofcom News Consumption Survey 2020 (Ofcom, p.56) and in Brazil, according to the Reuters Institute for the Study of Journalism (Digital News Report 2020, p.90).

**Fig. 1:**
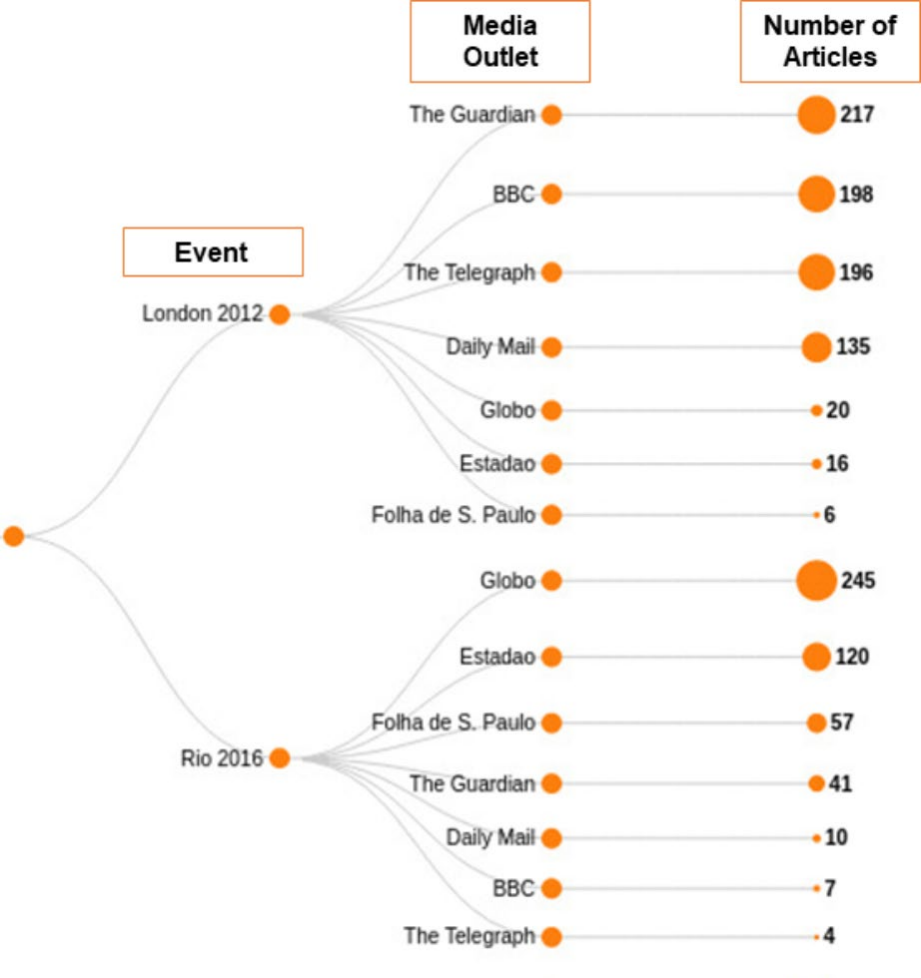
Distribution of news articles in the dataset. The number of articles in the corpus for Brazilian news media covering *London legacy* and British media covering *Rio legacy* are smaller than media outlets covering events hosted by their countries

The articles were collected using a Google search in 2020, the year when the next summer Olympics should have happened in Tokyo^6^. Using Google’s advanced search, a query was written stating the website (e.g. ‘bbc.co.uk’), the year and the keywords. Keywords used were *“Olympic legacy” London, “Olympic legacy” Rio, “Legado Olímpico” Londres*, and *“Legado Olímpico” Rio*. A list of URLs resulting from the search was then used to perform the web scraping, described in detail in the next section. The texts cover a period from 2004 to 2020. Their distribution over time is not homogeneous, being the year of each Olympics (2012 and 2016) the period with the highest number of articles. Also, news outlets published more articles covering the event hosted by their own country than the other one. The following figure illustrates the data distribution, starting by the event, followed by media outlets and the number of articles collected (Fig. 1).

## Data preparation

As the main purpose of this study is understanding the sentiment in the news covering two specific events, it was important to certify that the articles collected were focused on London 2012 and Rio 2016 Olympic legacies. First, documents needed to be cleaned to make sure that only the body text was being considered. Deleting clutter in web pages means identifying all peripheral information such as an advertisement that is irrelevant for this study and excluding it from the final list of stored articles. To do so, we first used *newspaper3k* (Ou-Yang, 2018) python library to scrape news text from the URLs obtained in the data collection step and then further scraped text from HTML tags where the python library failed. The python library specifically failed for news article pages from pre-2010 which had different html page structure and tags compared to the post-2010 article pages. To overcome this, we manually selected headline and body text tags from the pages to extract the content. Articles that do not mention the words ‘London’ and ‘legacy’ or ‘Rio’ and ‘legacy’ were also excluded.

A list of the articles was produced and they were manually labelled as ‘London’ or ‘Rio’. However, sometimes articles mention the Olympic legacies of London and Rio but not as the main topic. It can be, for example, an article about the Athens Olympics that mentions London 2012. However, in this case, the sentiments detected in the article would refer to Athens and not London as expected. For this reason, these articles had been identified and removed.

A few preprocessing steps were taken before the texts were ready for sentiment analysis. We used *spaCy* (Honnibal & Montani, 2017), a natural language processing library to segment the whole article into sentences with language-specific models for both English and Portuguese. Previous work on multilingual (Dashtipour et al., 2016; Zhang et al., 2018) and Portuguese text sentiment analysis (Cirqueira et al., 2016; Farias et al., 2016; Pereira 2021; Tavares et al., 2021) often use translation-based methods as baselines to compare against language specific approaches. As demonstrated by Araújo et al., (2016, 2020) and Cirqueira et al. (2016), machine translation from Portuguese into English significantly improves the sentiment results in comparison to the use of non-English classifiers. Having said that, previous work also refers to mistakes introduced into sentiment analysis method by translation to English language. To understand this better, for Portuguese news articles, we use *google-translate API* (Han, 2020) for translating text into English and evaluate different English sentiment classifiers.

As the words *Olympic* and *legacy* appear in the entire corpus and seem to carry positive sentiment, an alternative dataset was prepared by replacing different instances of *Olympic* by *event* and *legacy* by *outcome* to avoid possible bias. This word replacement was performed in both English and Portuguese texts, summary of which is provided in Table 1.

**Table 1:** List of words replaced to avoid bias in the corpus

Words	Replacement
Olimpíadas, olimpíadas, Olimpíada, olimpíada	evento
Olímpicos, olímpicos, Olímpico, olímpico, Olímpicas, olímpicas, Olímpica, olímpica	do evento
Legado, legado, Legados, legados, heranças, herança, Heranças, Herança	resultado
Olympic, Olympics, olympics, olympic	event
legacy, Legacy	outcome

## Evaluating the classifiers

To start our analysis, we had first selected four popularly used SA classifiers to apply to our corpus: SenticNet, SentiStrength, Vader and BERT. They are divided into three types: lexicon, lexicon and rule, and contextual based algorithms. SentiStrength (Thelwall, 2017) is purely lexicon-based, whereas SenticNet (Cambria & Hussain, 2015) and Vader (Hutto & Gilbert, 2014) are lexicon and rule-based algorithms. They split the text into words (termed as concepts) and look for their scores in the dictionary (Thelwall, 2014). They cannot disambiguate meanings as they do not always take the context into account. With rule-based algorithms, syntactic rules are applied over a sentence to get the final score between − 1 (Negative) and 1 (Positive). The implications of these characteristics will be discussed later on. For SenticNet, we contacted the authors and got access to the API, which provides the sentiment score (by applying rules) for a sentence. The publicly available SenticNet library only provides scores for words (concepts). For SentiStrength, scores are averaged of all the words (concepts) in the sentence to get the final score. These algorithms are available in English and Portuguese and were applied in texts written in both languages.

BERT, a contextual based algorithm trained in three different datasets, was also used in our corpus to get scores for texts in English and Portuguese. BERT^7^ is a machine learning model trained on a very large corpus of text such that it can encode words and sentences into vectors of real numbers while capturing the surrounding context of each word. As it is done in practice, these pre-trained models are fine-tuned (re-trained) for a target task, which in our case is sentiment prediction.

For English, we used two sentiment detection datasets which are publicly available: Amazon reviews (McAuley & Leskovec, 2013; Devlin et al., 2019) and tweets (Go et al., 2009) and trained two separate models named Amazon BERT and Sent140 BERT. To utilise BERT for detecting sentiment in Portuguese text, we use a publicly available Twitter corpus (Souza et al., 2020) and fine-tune Portuguese BERT for the sentiment detection task. These classifiers were used to assign sentiment labels to news headlines and the whole articles. For assigning a sentiment label to the whole article, we use the proportion of sentences of each sentiment type instead of averaging the sentence scores. With averaging scores, there is a chance of negative and positive scores cancelling each other and the final score being closer to zero (Neutral). Proportion of sentences with a particular sentiment type provides a much clearer view of the narrative used in the article. For example, if the proportion of sentences with negative labels is greater than 50%, then the article is assigned a negative label.

**Chart 1:**
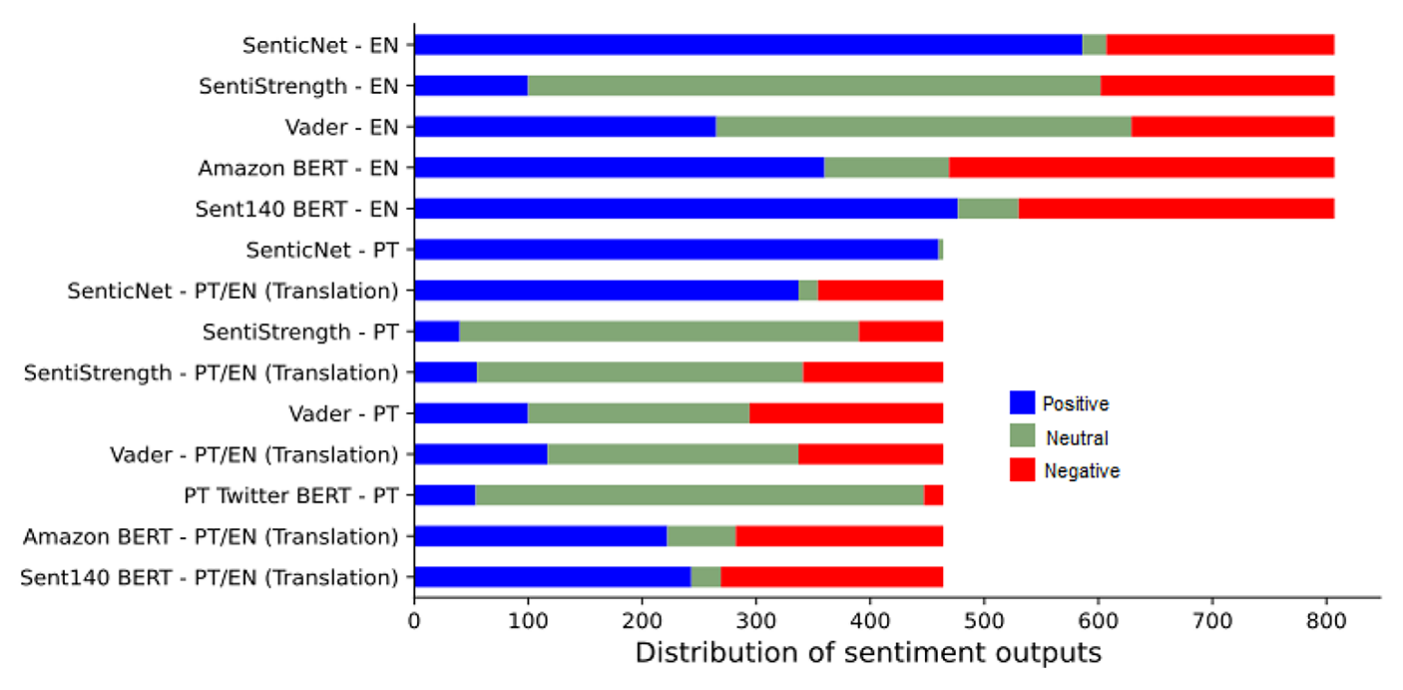
- Distribution of models outputs (positive, neutral and negative per classifier for data originally in English, data originally in Portuguese and data translated from Portuguese into English. Data in Portuguese contain 464 articles and data originally in English contain 807. PT/EN means Portuguese text translated to English

Chart 1 shows the distribution of positive, neutral and negative sentiment labels produced by each classifier for data in English and Portuguese.

To evaluate the performance of the algorithms, both the sentiment expressed by the news articles and the news headlines of four outlets - two in English and two in Portuguese - were labelled by one of the authors who has been studying these specific events for two years. As a domain expert, he assigned positive, neutral and negative labels to 717 news articles (56.4% of the total number of articles)^8^. This list of what we called *gold labels* was used as a reference to compare with the results generated by each classifier, in order to evaluate their accuracy for this specific dataset. News headlines were chosen as they intend to summarise the article’s content in one sentence. Table 2 describes the distribution of positive, neutral and negative sentiment classifiers outputs for each of the four clusters, as well as the distribution of *gold labels*.

**Table 2:** Distribution of positive, neutral and negative sentiment outputs for each cluster used to analyse classifiers accuracy, followed by *gold labels* distribution

	News outlet/Event											
	Globo (Rio)			Estadao (Rio)			Guardian (London)			Daily Mail (London)		
	Pos	Neu	Neg	Pos	Neu	Neg	Pos	Neu	Neg	Pos	Neu	Neg
**PT SenticNet**	243	2	0	119	1	0	x	x	x	x	x	x
**PT SentiStrength**	20	184	41	15	89	16	x	x	x	x	x	x
**PT Vader**	57	103	85	25	49	46	x	x	x	x	x	x
**PT Twitter BERT**	7	234	4	13	104	3	x	x	x	x	x	x
**EN SenticNet**	179	8	58	92	3	25	167	1	49	90	4	41
**EN SentiStrength**	26	151	68	19	69	32	23	149	45	24	67	44
**EN Vader**	69	110	66	26	56	38	61	107	49	44	56	35
**EN Amazon BERT**	140	21	84	45	21	54	94	33	90	52	16	67
**EN Sent140 BERT**	135	11	99	57	8	55	144	11	62	65	12	58
**Gold Labels**	79	64	102	32	28	60	93	55	69	44	26	65
Total Titles	245			120			217			135		

The scores, converted into three labels (positive, neutral, negative), were compared with the *gold labels*. The results of this comparison, for news headlines, are shown in Table 3.

**Table 3:** (%) Matches of each classifier with the gold labels (accuracy evaluation for this dataset) for news headlines. PT: Portuguese; EN: English. Data distribution (number of articles): Globo (Rio): 245; Estadao (Rio): 120; Guardian (London): 217 ; Daily Mail (London): 135

Cluster (Outlet/Event)	PT SenticNet	PT SentiStrength	PT Vader	PT Twitter BERT	EN SenticNet	EN SentiStrength	EN Vader	EN Amazon BERT	EN Sent140 BERT
Globo (Rio)	33%	39.5%	49.7%	24.5%	46.5%	50.2%	55.5%	55.5%	61.2%
Estadao (Rio)	26.4%	15.7%	39.6%	3.3%	38.8%	31.4%	40.4%	53.7%	57%
Guardian (London)	x	x	x	x	48%	49.6%	56.2%	60%	58.5%
Daily Mail (London)	x	x	x	x	52%	47.9%	56.6%	61.7%	61.2%

The table shows how many matches were found when analysing news headlines and comparing the labels produced by each of the sentiment classifiers with the *gold labels* assigned by the expert. As expected^9^, the English translation performed much better than the original text in Portuguese. Among all classifiers, the best results were obtained from Amazon BERT and Sent140 BERT - around 61% for Daily Mail news titles covering the London 2012 Olympics.

## Combining classifiers

After running different classifiers in the same texts, the wide variety of outputs raised questions on the value of these results for the planned quantitative analysis presented above. With the purpose of improving the percentage of matches with the *gold labels*, and therefore our trust in the results, four approaches combining different techniques were tested. The purpose was achieving more matches with a combination of classifiers than when using only Sent140 BERT alone. This combination would correspond to the method of using different people to assign sentiment labels to a sentence in order to train a module. The tests are presented below.

(A) Combination of five sentiment classifiers (majority agreement).

This approach consisted of combining the labels produced on news headlines with the five algorithms for data in (original and translated) English (SenticNet, SentiStrength, Vader, Amazon BERT and Sent140 BERT). If three or more algorithms agree among them - assigning the same label -, then this label was considered as correct. If less than three of the algorithms agree, a label ‘inconclusive’ was assigned to the contradictory result.

### Result of approach A

The result was worse than Sent140 BERT applied alone. For Daily Mail covering London, the matches dropped from 58 to 53%. The problem was that SenticNet and SentiStrength were less accurate than the others so they impacted the result negatively.

(B) Combination of top three sentiment classifiers (discarding two worst scores).

This approach ignored the two classifiers with worst scores detected in comparison with the *gold labels*, using the remaining three: Vader, Amazon BERT and Sent140 BERT. If two of them agreed with the same label, then this label was considered as right. Otherwise, the label ‘inconclusive’ was assigned again.

### Result of approach B

Although better than approach ‘A’, the results were almost the same as those produced using just Sent140 BERT. For Globo covering Rio, the matches increased only 1%. The issue was the number of ‘inconclusive’ labels assigned.

(C) Combination of *three classifiers* replacing *inconclusive* by Sent140 BERT.

Still using approach ‘B’, but where agreement was *inconclusive*, we fallback to the label assigned by the best classifier: Sent140 BERT, as determined at the start of testing.

### Result of approach C

The results were slightly better than Sent140 BERT alone. For The Guardian covering London the matches increased from 61 to 70%. However, there were many inconsistencies. When we replaced *inconclusive* by Vader or Amazon BERT, instead of Sent140 BERT, the final result in percentage was very similar, even though they produced different labels for the same texts. Results of approach ‘C’ were more of a coincidence than accuracy.

(D) Combination of *three classifiers* ignoring *inconclusive* sentences.

The last approach consisted of calculating the number of *inconclusive* labels assigned by approach ‘B’ and ignoring them in the analysis. The number of *inconclusive* titles varied from 7 to 17% among the four outlets used for testing. It is very hard to assign consistent labels to these sentences in which the three algorithms disagree: each of them assign a different label to the same sentence. In this case, instead of trying to produce a new label, these sentences were considered as impossible to detect sentiment and ignored in the quantitative analysis. They can be later closely read to identify their particularities, but they do not contribute to the purpose of the quantitative approach.

### Result of approach D

The number of matches increased significantly as only those sentences where at least *two classifiers* agree in assigning the same label were used. For The Guardian covering the London Olympics, matches increased from 61% using Sent140 BERT to 74% using approach ‘D’. This approach was applied to the rest of the dataset to provide better data for quantitative analysis.

Table 4 summarises the results of each approach, presenting the number of matches between the results found using each approach and the *gold labels*.

**Table 4:** Accuracy of each approach for news headline text (%)

Method	Globo (Rio)	Estadao (Rio)	Guardian (London)	Daily Mail (London)
Sent140 BERT	61%	57%	61%	58.5%
Approach A	59%	48.3%	59.4%	53.3%
Approach B	62%	57.5%	66%	57.7%
Approach C	65.3%	59.16%	70%	61.4%
Approach D	66.9%	65%	74.7%	69.6%
Inconclusive	7.3%	11.6%	10.5%	17%

Results of approach ‘D’ indicate that using only the current state-of-the-art sentiment classifier corresponds to ignoring contradictory evidence from other algorithms. Therefore, what this method offers is the identification of contradictory cases that influence the amount of inaccurate outputs produced by classifiers.

In the following sections, the issues and results of the application of approach ‘D’ are discussed. The main objective was to understand what kinds of insights about the media coverage of the Olympic legacy of London 2012 and Rio 2016 this method provides.

## Results

The results discussion will be divided into two sections. In the first one, issues encountered while working with the method, such as translation problems, were presented. In the second section, the results were applied to the case study bringing out an analysis of the sentiment expressed by the media in the coverage of the two events.

## Section 1: potentialities and limitations of the method

As pointed out by Rogers (2019, p.3), the process of recognising problems with the method and the data is inherent to the work with digital methods. Working with sentiment analysis according to approach ‘D’, proposed by this paper, culminates in some inconsistencies that are discussed in this section.

First, it is important to point out the disagreements between the expert and the machine. For the headlines *Martin Samuel: Tessa Jowell deserves an Olympic medal in utter madness* (Samuel, 2010), *Olympic legacy: school sports provision patchy across UK, admits Jeremy Hunt* (Press Association, 2012) and *Letters: The true Olympic legacy is white elephants on our doorstep* (Pimm et al., 2012) the tool labelled them as positive utterances while the expert labelled as negative. It is hard to explain why the machine assigned some labels to specific sentences, but there are mechanisms to hypothesise the cause.

One of the reasons why models make such mistakes can be attributed to the domain gap, which is the difference in the domain of training data - such as primarily social media posts - versus the evaluation data - as news headlines and text, for example. Cross-domain sentiment analysis is a challenging and fairly active research area (Al-Moslmi et al., 2017). While many techniques exist to mitigate the problem, most require a variant of cross-domain learning using huge source data, with labels, and target data - such as news. The objective of the learning process is to get a better performing sentiment detection model for the target data. In this work, we rely on the existing publicly available monolingual sentiment detection models and investigate the model outputs with a fine-grained analysis.

**Fig. 2:**
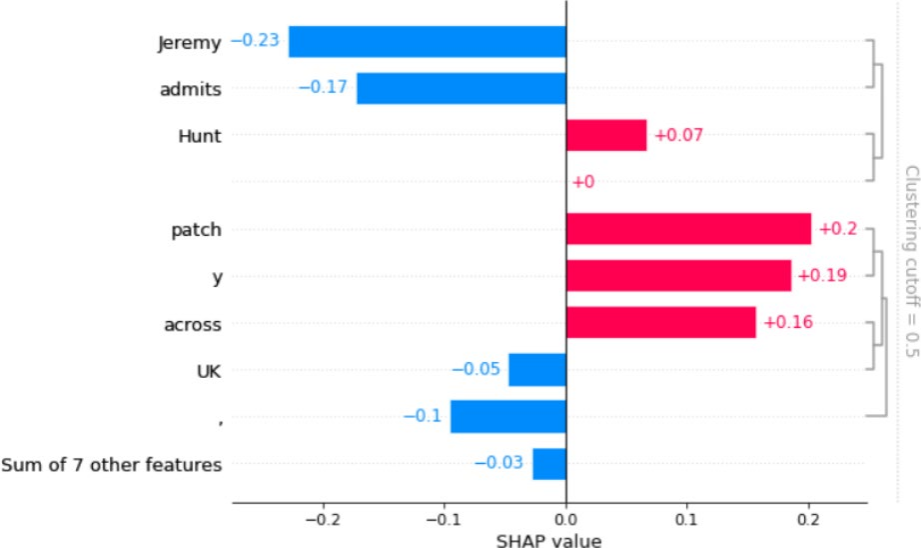
- Shap returns a pictorial depiction of Shapley values. Only the words considered as having significant contribution to the final classification are shown. The negative sentiments are represented in red. The positive, in blue. The label assigned to this sentence by the combination of classifiers was *positive*

To hypothesise on how the algorithms work, we have used explainable AI, a mechanism to unveil the machines behaviour (Linardatos et al., 2021). We have used the tool named SHAP (Lundberg & Lee, 2017) on the two best performing classifiers: Amazon BERT and Sent140 BERT. The tool used Shapley values to depict how fairly the outcome can be distributed among the different features. Thus, the Shapley values of every feature signal its contribution for a given predicted (into negative, positive or neutral) instance. We utilised an existing tool to quantify the feature importances for BERT-based models. The input to the tool is a text and the classifier. Shap returns a pictorial depiction of Shapley values for every feature (Fig. 2), which are words in this case. The output enables easy examination of the predictions. Figure 2 illustrates SHAP visual representation for the news headline ‘Olympic legacy: school sports provision patchy across UK, admits Jeremy Hunt’.

This visual representation provides information on the role each word plays in the sentence’s sentiment output. By looking at Fig. 2, we could identify the words that added a higher score of negativity to the headline such as ‘patchy across’, and those which added more positivity such as ‘admits Jeremy’. We ran this example again but deleting this time the words ‘admits Jeremy Hunt’. This new experiment resulted in a strong negative sentiment output that looked more coherent to the sentiment expressed by the news title. Entities like ‘Jeremy Hunt’ are one of the several reasons why sentiment is misinterpreted by sentiment classifiers. Below, we list the cases where the tool and the *gold labels* disagree, categorising the mistakes according to its nature. This list works as a recommendation for researchers to consider when using SA to interpret texts.

- **Entity**: As in *Olympic legacy: school sports provision patchy across UK, admits Jeremy Hunt* (Press Association, 2012), entities carry sentiment. Sometimes, their sentiment is relevant to the analysis. However, this is not the case for this sentence as Jeremy is not the main subject of the headline but ‘school sports provision’. Jeremy here added positivity, which had wrongly influenced the final sentiment output. To come to this conclusion, we have replaced Jeremy Hunt with the names of two other British politicians mentioned in the corpus: Boris Johnson and Ken Livingstone. Despite the positivity of the word ‘admits’, both Johnson and Livingstone added significant negativity to the final score.
- **Syntax**: For this example *The Essential Morning: the melancholy inheritance of the Olympics* (Globo, 2019), the sentiment output was positive, although this is clearly negative. The problem is that the first part of the sentence ‘The Essential Morning’ adds positivity to the final score. This is the name of the newspaper’s section where the news of the day is summarised in the mornings. Therefore, the first sentence is not part of the main topic covered by the news article. Two or more sentences separated by punctuation can cause some inconsistencies like this.
- **Semantic**: In *Environmental legacy, the great debt of Rio Olympics* (Soares, 2016), although ‘debt’ was correctly assigned a negative label, ‘great’ was misunderstood. It was read as an adjective meaning ‘good’ instead of an adverb meaning ‘big’. This mistake added high positivity to the sentence, influencing the final result.
- **Negation**: Negation is a huge challenge to NLP tools (Kassner & Schütze, 2019). In *Olympic stadium will not be white elephant after London 2012* (Gibson, 2011) the word ‘not’ changed the meaning of the rest of the sentence to its opposite. Some models not only ignore this feature but also give to the word ‘not’ a high negative score, which provides improper classification to the text.
- **Metaphors**: White Elephants are among the most representative negative aspects of the Olympic legacies (Leopkey & Parent, 2012). It usually refers to those infrastructures constructed for the games that were abandoned right after the event or remain in a precarious condition not serving for the purpose it was initially planned. It however was not classified as negative by the algorithms in *Letters: The true Olympic legacy is white elephants on our doorstep* (Primm, Wood & Rose, 2012).
- **Domain-specific words**: In the title *Britain’s Olympic legacy is a sedentary nation* (Conn, 2015), the word ‘sedentary’ carries a specific negative connotation. In the context of Olympism, a sedentary nation means that people are not playing sports as desired by the legacy plan. It means that the plan fails to inspire local citizens to be more active. In this example, however, the word sedentary was assigned with a positive label.
- **Sarcasm**: As even humans struggle to identify sarcasm, for machines the task is even harder (Farias & Rosso, 2017). In this news headline *Martin Samuel: Tessa Jowell deserves an Olympic medal in utter madness* (Samuel, 2010), the expression ‘deserves an Olympic medal’ was classified as highly positive, ignoring the sarcasm in the sentence.
- **Objective statements**: For sentences like *London 2012 Olympics will cost a total of £8.921bn, says minister* (Gibson, 2012), it was difficult to assign a label as the sentiment of the word ‘cost’ varies depending on the context. A cost lower than expected is positive, for example. However, words like ‘cost’ and ‘pay’ were mostly classified as negative by the algorithms.

A last point to be mentioned regarding the use of sentiment analysis techniques is the effect of translating texts originally written in Portuguese into English.

## Impacts of translation

As discussed above, the use of SenticNet, SentiStrength, Vader and BERT to classify Portuguese text has produced unsatisfactory results. Working with data in Portuguese has then required automated translation into English. Although widely used and recommended in some cases for non-English SA (Araújo et al., 2016, 2020), machine translation impacts the scores and can lead to misreadings (Dashtipour et al., 2016). However, Balahur & Turchi (2012) evaluated the impact of machine translation from German, French and Spanish into English, with two being romance languages like Portuguese, and concluded that the noise exists, but the result is still reliable.

**Chart 2:**
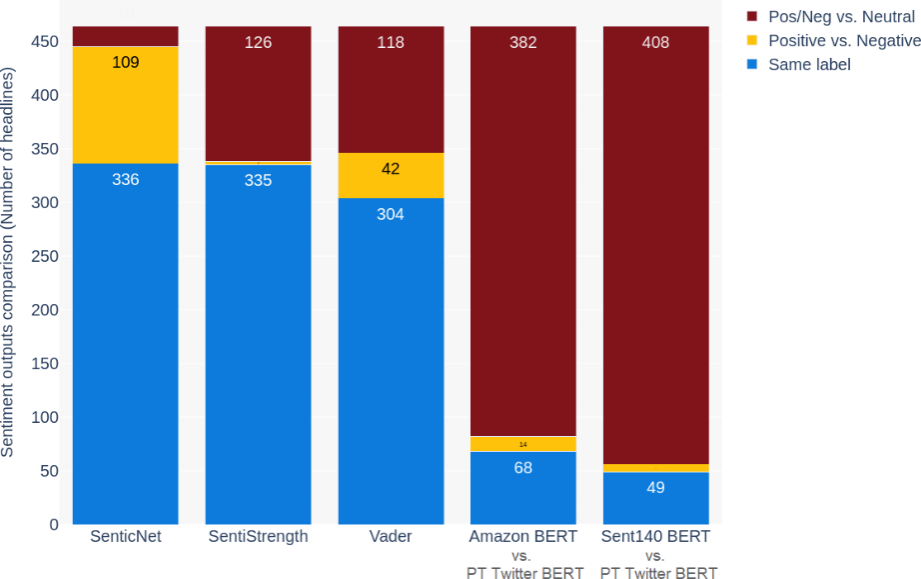
- Variation of sentiment in the 464 news titles originally published in Portuguese by the Brazilian press and then translated into English

Chart 2 shows the comparison between labels produced using the four classifiers for the original news titles in Portuguese and the translated version in English. BERT trained in Amazon and tweets datasets were compared individually with Portuguese Twitter BERT. The main purpose is to observe how often translation changes the sentiment of the sentences. The chart represents a total of 464 articles published in Portuguese divided into three sections. Blue represents the number of titles that kept the same label in both languages. In yellow, are shown those titles whose sentiment changed to the opposite direction: from positive to negative or from negative to positive. On the top of the bars in red are those titles whose sentiment varied slightly from positive or negative towards neutral, for example.

According to chart 2, SenticNet and SentiStrength resulted in around 72% of agreement between the label assigned to the original and translated version of the sentences. Vader presented slightly less matches, with 65% of the titles labelled the same. It is interesting to highlight the amount of titles whose sentiment varied to the opposite label, in yellow. SenticNet presented the highest disagreement around 23%, followed by Vader with 9%. Amazon BERT and Sent140 BERT presented the highest variation towards neutral results. This occurred because most outputs from Portuguese Twitter BERT were ‘neutral’, as shown in chart 1.

This experiment allowed the conclusion that SentiStrength presented the best equivalence between Portuguese and English translated version, presenting no change in 72%, slight variation in 27% and less than 1% of opposite results. The reason for this is that, as a lexicon based algorithm, it outputs a similar score for words in both languages, while the others are affected by rules that take the syntax into consideration, producing different results. However, due to the lower efficacy of SentiStrength discussed above in the methodology, this classifier was not used in the approach ‘D’. Nevertheless, this experiment provides a better understanding of the impact of the translation in Vader, one of the three classifiers selected for this study, and potentially other classifiers which rely on syntactic cues.

By looking closely at some of the sentences translated into English, some issues could be identified. In Table 5, there is an example of error in the first two headlines in the translation of the word *corte*. *Corte* in Portuguese can be both the verb *to cut* or the noun *court* in English. The first sentence should have translated the verb as *cut* and the second as *court*.

**Table 5:** Comparison between the original sentences in Portuguese and their translations into English

Portuguese (original)	English (translation)
Brasil anuncia ao COI corte de R$ 900 mi no orçamento dos Jogos	Brazil announces the COI Court of R$ 900 mi in the games budget
Legado olímpico virou milhões em propina a ‘amigos da corte’ de Cabral, diz MPF	Olympic legacy turned millions in tipping ‘Cut Friends’ of Cabral, says MPF
Olimpíada é ‘desculpa fantástica’ para mudar o Rio, diz prefeito	Olympiad is ‘fantastic excuse’ to change the river, says mayor

The third sentence shows a mistake in the translation of the word *Rio*. It should remain *Rio* as the name of the city *Rio de Janeiro* and not translated into *river*, the meaning of the word *rio* in Portuguese. These mistakes have impacted the sentiment of the news headlines and reveal what sort of issues can be faced by researchers when using this technique to explore large amounts of automated translated texts. This highlights the need for the development of more tools that work in languages other than English.

This methodological discussion was fundamental for the development of the case study analysis, as it provided us with a better understanding of what kind of data we are analysing. Taking these issues into consideration, the following section is focused on the interpretation of results achieved with the use of the technique developed in approach ‘D’ in the news articles covering the Olympic legacies of London 2012 and Rio 2016.

## Section 2: the sentiment in the news coverage of the legacies

**Chart 3:**
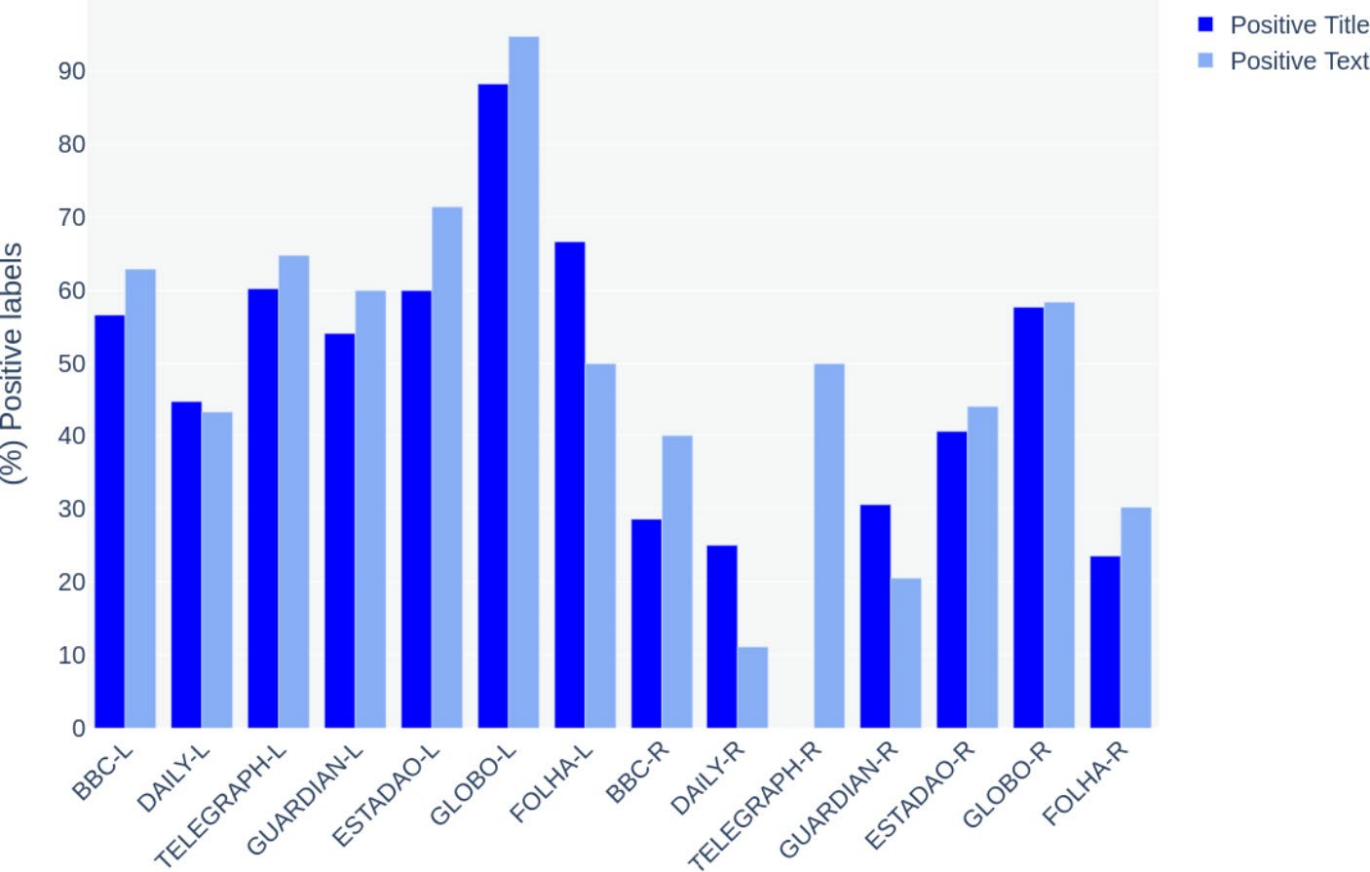
- (%) Comparison between the percentage of positive labels assigned to news titles versus body text

**Chart 4:**
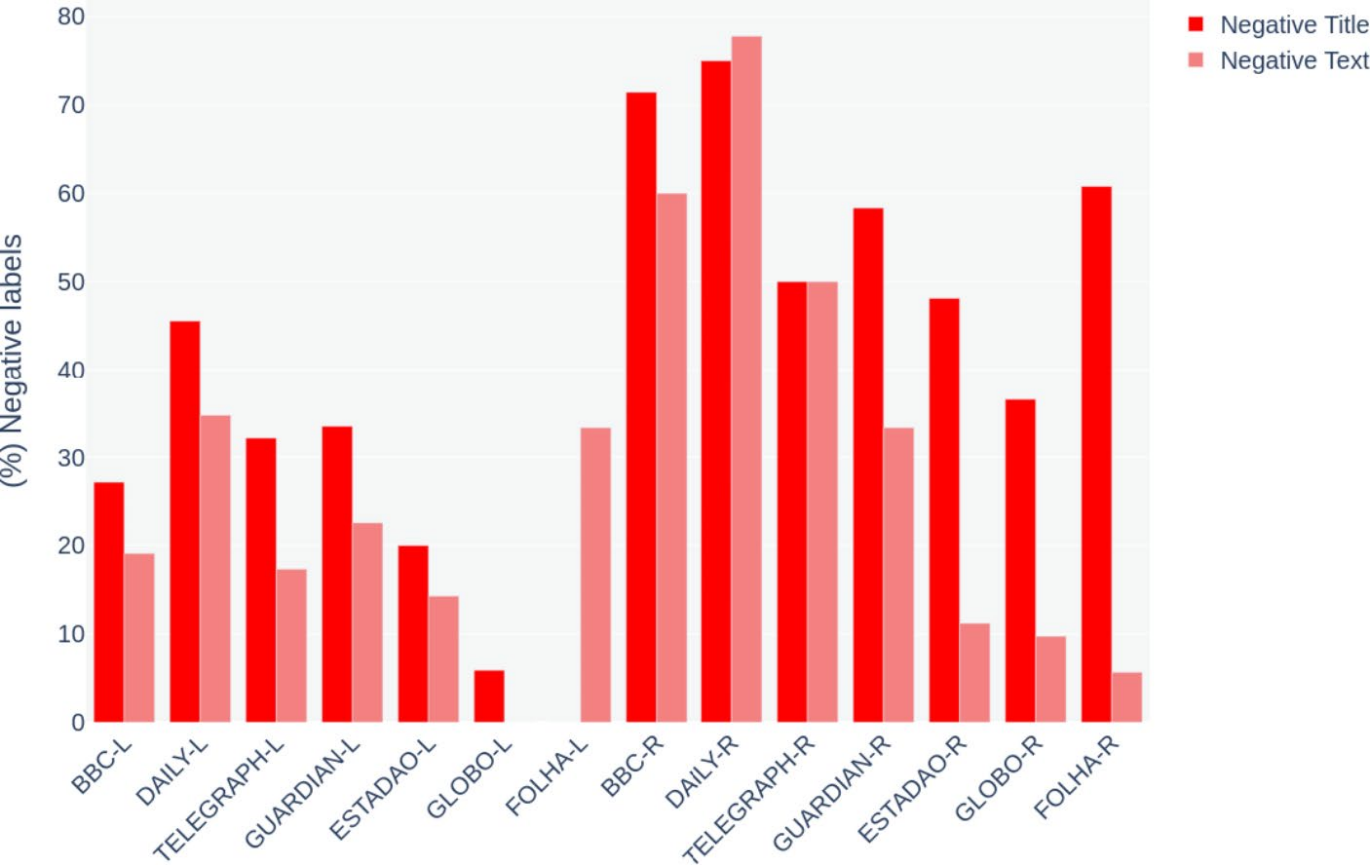
- (%) Comparison between the percentage of negative labels assigned to news titles versus body text

By plotting the labels produced with approach ‘D’ in charts, we had an overview of the results. Charts 3 and 4 illustrate the percentage of positive and negative labels assigned to news titles and body texts. The name of the outlets is followed by letters that indicate the event they cover: *L* for London and *R* for Rio.

Charts 3 and 4 show that news titles tend to be less positive and more negative than their respective articles. Considering the particularities of the text type used as data here, titles have fundamental importance. What today is often referred to as ‘Clickbaits’ (Scacco & Muddiman, 2016) in online news is a result of an old strategy widely spread in the newsrooms. Rayner et al., (2001, p.227) describe news headlines as ‘hooks’ aiming to grab the eyes of the audience. For the authors, however, more than call the attention, news titles must keep a certain level of mystery, creating a problem to be solved in the body text.

Although not recent, this logic of convincing the reader to buy the story becomes central to the production of online news whose audience is calculated based on the number of visitors and clicks received by the website. Many online newsrooms assign to a ‘homepage editor’ the function of producing creative headlines to make the content more attractive. Titles are key for the analysis of news articles (Piotrkowicz et al., 2017) as they are responsible for summarising the main subject to be addressed by the text but also because they make use of adverbs and adjectives such as the already mentioned ‘huge disappointment’ to emphasise and qualify the content. This strategy allows for a better use of sentiment detectors providing better insights on the broad sentiment of the news coverage.

**Chart 5:**
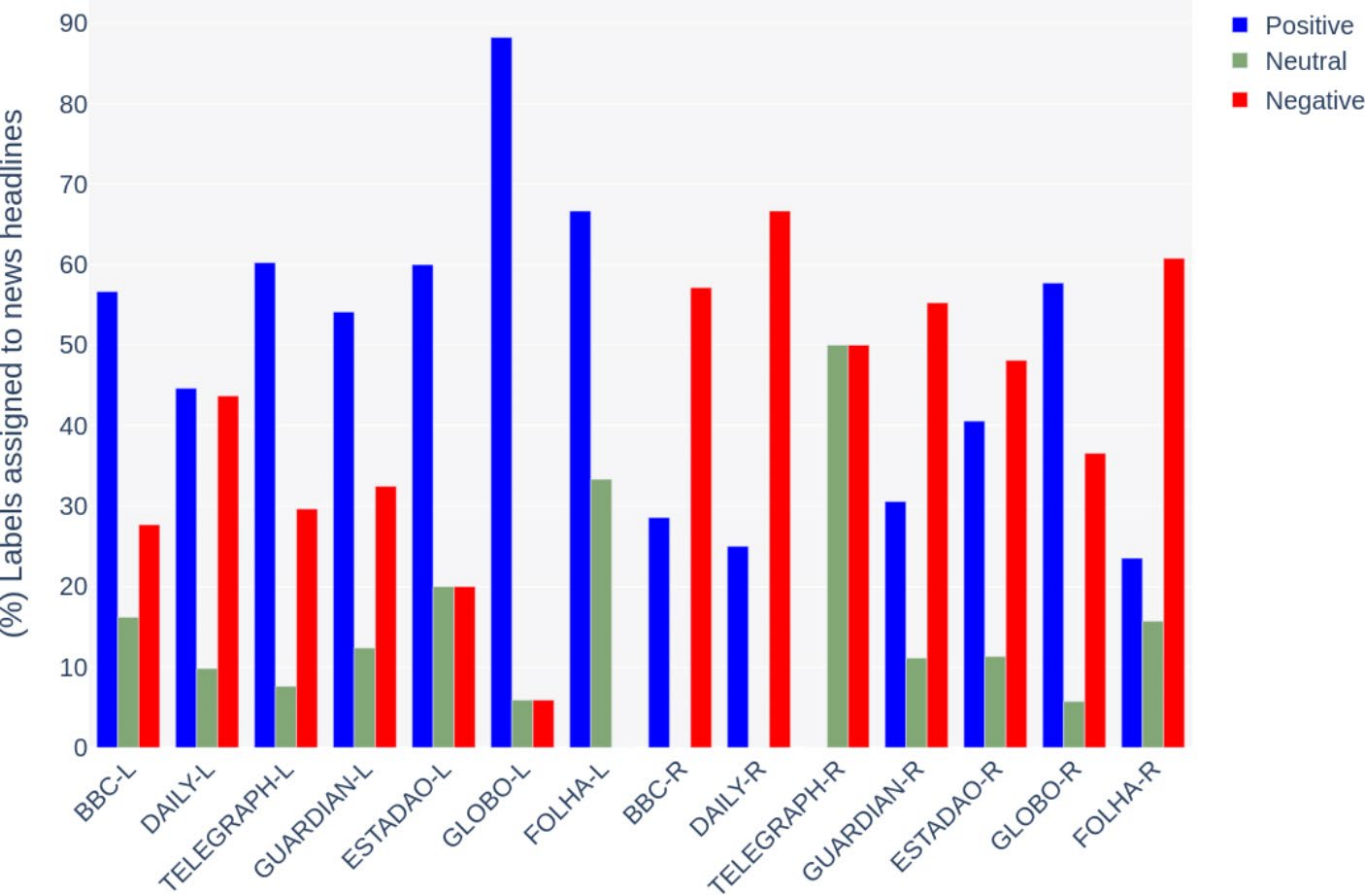
- (%) Comparison between the percentage of positive, negative and neutral labels assigned to news titles

**Chart 6:**
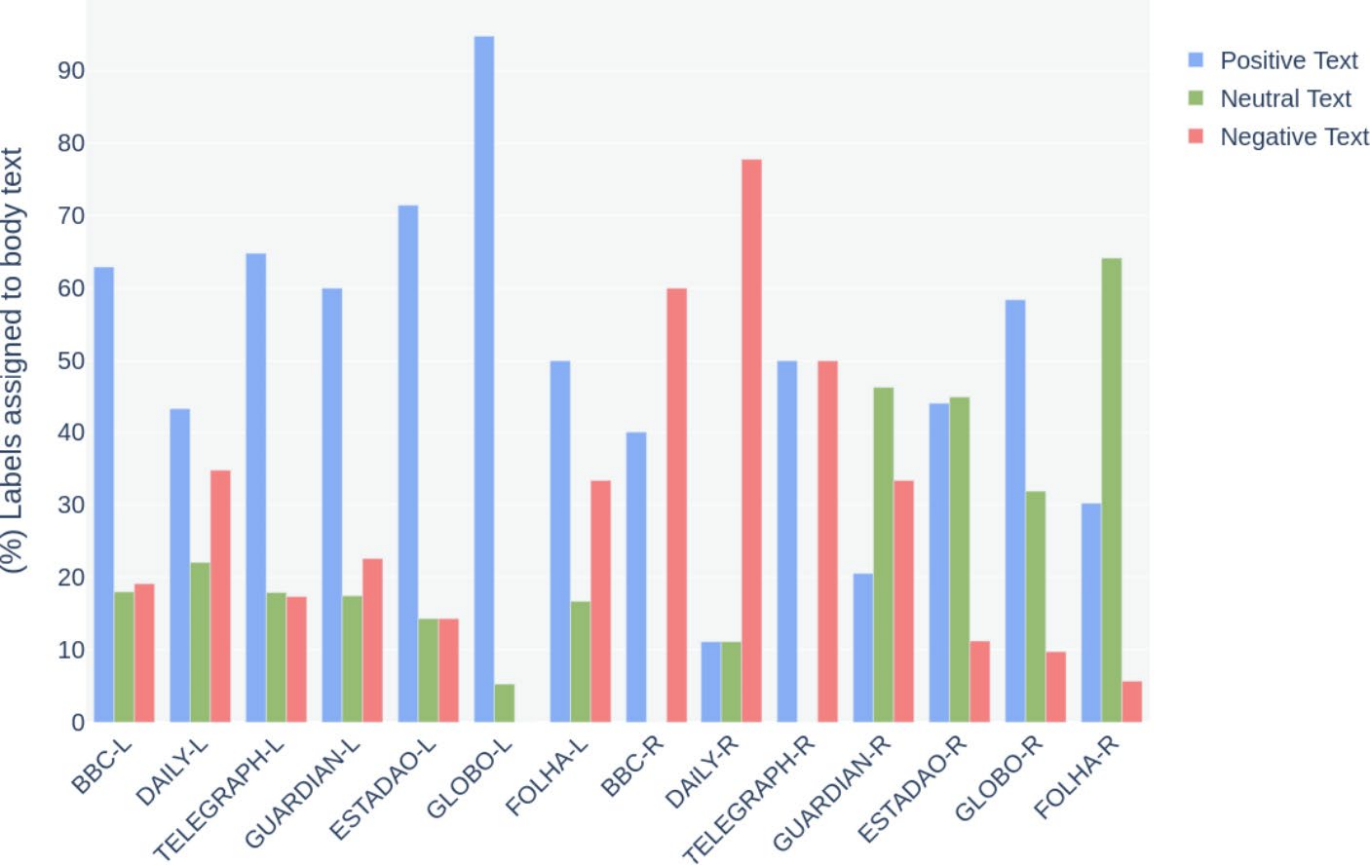
- (%) Comparison between the percentage of positive, negative and neutral labels assigned to body text

Corroborating to this understanding, chart 5 shows how positive and negative labels are assigned significantly more to news headlines than neutral ones. Chart 6 illustrates the sentiments of the body texts, where the number of neutral labels increased, especially for the last four outlets: The Guardian-R, Estadao-R, Globo-R and Folha-R. Although titles for these outlets were more negative, the sentiment of the articles’ body text did not correspond to the titles.

By comparing news headlines’ sentiments, it is evident the contrast between the two events: the media coverage of London’s legacy was significantly more positive, while Rio’s was more negative. Regarding London, the British media has been more critical than the Brazilian. Estadao, Globo and Folha presented a very low number of negative headlines when referring to the London 2012 Olympics. However, when covering Rio, the British media has shown a higher level of negative news titles that correspond or, sometimes, are even more significant than the local Brazilian outlets.

## Interpreting sentiments

The analysis of these sentiment outputs, combined with a qualitative close reading approach, reveals how the narratives are embedded in a utopian-dystopian dichotomy. As stated by Girginov (2018), the concept of Olympic legacy is a social construction that promotes ‘specific visions of what the desired development should look like’. (Girginov, 2018, p.196). It is an idealised form of legacy that we refer to when using the term utopian narrative. This phenomenon is particularly promoted by official government communication strategies that emphasise the catalyst potential of the event for urban development.

On the other hand, the fear of not achieving the promises, evidenced by the use of words such as ‘white elephants’, narrates a chaotic effect of the Games. This dystopian idea became prominent in expressions used by authors like Zimbalist (2017), who initiated his book’s introduction about the Rio Olympics with the sentence ‘welcome to hell’. Based on this paper’s analysis, we concluded that there is an overwhelmingly positive view of the Brazilian media in relation to London 2012, while the British media has primarily focused on the negative aspects of Rio’s legacy, silencing other perspectives.

## The scepticism of Londoners and the optimism of brazilians

Plotting the results in a chart divided into three parts representing the sentiments before, during and after each event facilitated the analysis of how positive, neutral and negative results varied overtime.

**Chart 7:**
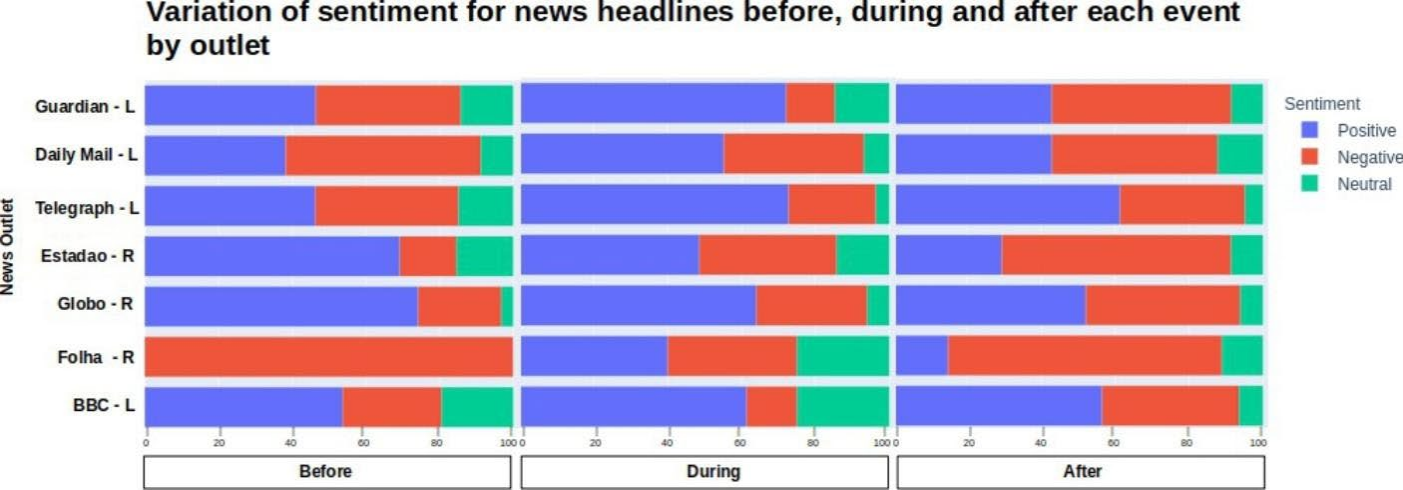
- (%) Variation of sentiment for news headlines before, during and after each event by outlet. Timeframe for London 2012 Olympics: before (2004 to 2011), during (2012), after (2012 to 2020). Timeframe for Rio 2016 Olympics: before (2009 to 2015), during (2016) and after (2017 to 2020)

There are some important characteristics of each of the three time periods mentioned above that must be taken into account. ‘Before’ contains mostly the expectations for the Olympics. News covering the planning and delivery of the first sports facilities were in focus. However, besides shedding light to the preparation, news also predicted possible effects of the Olympics as in *Olympic ‘legacy’ expected in Kent* (BBC, 2005). ‘During’ is composed of news covering the delivery of the first facilities as in *Olympic legacy: Rio community wins volleyball training centre* (Globo, 2016), as well as issues faced by the local organising committee to finish things on time. ‘After’ contains those articles that review the promises and compare with the present as soon as the event finishes, as in *After five months, Olympic legacy presents more negative points than positive* (Estadao, 2017).

By looking at the British media covering of London in Chart 7, it is evident the increase in positivity during the event in comparison to before it. The euphoria promoted by the Games impacted significantly the news, that were less critical while the event occured. After, however, the number of positive news titles dropped and negative raised.

For the Brazilian media there is a pattern followed by Globo and Estadao. Both presented a higher number of positive headlines before Rio 2016 that has reduced over time while the negative ones increased significantly. Folha, however, presented an anomaly, being only negative before, a bit more positive during and very negative again after the event.

The variation of sentiment over time points to an anaesthetising effect in the press during the Games. The agenda is impacted by the volume of news about sports, being the legacy a topic to be taken up later after the event cools down.

Comparing the behaviour of the media with the public opinion, we found a similar pattern. A survey conducted by Sesc RJ/FGV Projetos two months before the Rio Olympics has shown that more than 60% of Rio citizens believed in the success of the event (Galdo, 2016). Another survey applied a year later in 2017 by Datafolha (Folha, 2020), however, has revealed that 70% of Rio citizens evaluated the Olympic legacy as negative.

Regarding the 2012 Olympics, although two-thirds of Londoners said that they expected to pay for the additional costs of hosting the Games, 69% supported the event (BBC, 2006). The number changes significantly, however, when the broader national scenario is observed. Around 64% of the British public responded to a poll saying that although they see the good impacts of the event for London, they do not see the same for themselves (Gardiner, 2012; Dolan et al., 2016) compared the Londoners’ sentiments after the Games with Parisians and Berliners and concluded that London citizens were significantly happier during the Games than the others, but that this sentiment returned to normal levels in the year after. As pointed out by Hiller & Wanner (2018), the measurement of the public opinion about the Games is impacted by a diversity of sentiments that coexist and transform over time, which makes its study challenging. However, looking at these surveys can be informative for the study of the news sentiment as similar patterns were identified by comparing both results.

## Conclusion

This study has looked at the use and combination of multiple SA algorithms to explore news articles published in English and Portuguese covering the Olympic legacy of London 2012 and Rio 2016. First, we have developed a methodology that started by evaluating and combining four SA algorithms to improve the quality of outputs. The experiments have shown that applying Vader, Amazon BERT and Sent140 BERT to the text corpus and extracting the agreements between at least two classifiers as a correct answer has considerably increased the accuracy of SA results for this specific data. This method was particularly important to identify contradictory SA outputs that, once removed, improved the level of certainty.

The lasting results were confronted using explainability tools and their limitations taken into consideration in the interpretation of the data. The results were divided into two sections. In section one, the behaviour of classifiers was analysed using Shapley to indicate possible reasons for the outputs, especially where the machine and the human expert disagreed. Reasons for the disagreements were identified, categorised and compiled in a list, which can be used by other researchers interested in using SA outputs to interpret sentiment expressed in texts. We have particularly looked at the impacts of translation and the challenges of working with multilingual NLP. This discussion provided information to be considered in studies seeking to apply SA techniques particularly to languages other than English. Texts in Portuguese were translated into English for the use of SA due to the poor performance in texts in that language. Although mistakes of translation had impacted the final sentiment classification, the overall outputs were considerably better than the use of any of the listed modules for Portuguese.

In section two, we have looked at the sentiment outputs as a tool to analyse the media coverage of the Olympic legacies. According to the results, news headlines presented more positive or negative labels, having a tendency to be more negative than their respective texts. When looking specifically at texts, neutrality increases significantly. These results are informative regarding the journalistic practises of using titles, especially online, to catch the attention of the audience.

By comparing the coverage of the two events, we concluded that London received a more positive coverage while Rio a more negative one. While the Brazilian media has been less critical about London, the sentiments expressed by the British media about Rio were very negative. We have referred to this phenomenon as a utopian-dystopian dichotomy, where one event is represented as a ‘huge disappointment’ while the other one as a ‘success’, silencing about or reducing the space in the agenda for the nuances embedded in these complex media events.

At the end, by looking at the variation of sentiment over time, we have identified that the British media was more sceptical about the legacy of the Games, while the Brazilian media was more optimistic. These results follow a similar pattern when compared with the public opinion about the events published in surveys before and after each Olympics.

Although challenging, the application of SA to news articles has revealed interesting aspects of the media event analysed. For future work, it would be important to compare how the limitations presented could be potentially minimised and how domain specificity can be addressed in the models.

## Data Availability

The dataset with sentiment annotation is published and available on https://github.com/caiocmello/sentiment-annotation-olympic-news.

## Footnotes

3 Beijing (China) and Almaty (Kazakhstan).

4 Paris (France) and Los Angeles (USA). Hamburg (Germany), Rome (Italy), Budapest (Hungary) and Boston (USA) withdrew their bid.

5 Brazilians outlets were initially four (Globo G1, O Globo, Folha and Estadao). Globo G1 is the news website of the Globo broadcast company. O Globo, however, is a newspaper that is part of the Globo group. For having similar editorial alignments, they were gathered in just one called simply ‘Globo’ in this paper.

6 The event was postponed to 2021 due to Covid-19 pandemic restrictions.

7 BERT: Bidirectional Encoder Representations from Transformers is a machine learning technique for NLP developed by Google.

8 The dataset is published and available on https://github.com/caiocmello/sentiment-annotation-olympic-news.

9 Araújo et al., (2020); Pereira (2021) and De Freitas & Vieira (2015) discussed the limitations of working with data in Portuguese.

## Code Availability

Not yet publicly available.
